# Correlation analysis of feeding intolerance and defecation after primary anastomosis for neonatal intestinal atresia

**DOI:** 10.1007/s00383-023-05603-x

**Published:** 2023-12-22

**Authors:** Ling Zhou, Yang Chen, Zhiyong Wang, Dongdong Chu, Dong Xiao, Ledao Zhu, Aihui Guan, Qianghui Liao, Jiashu Liu, Jiahui Li, Feng Ren

**Affiliations:** Shenzhen Children’s Hospital, Shenzhen, China

**Keywords:** Congenital intestinal atresia, Neonates, Postoperative feeding intolerance, Defecation

## Abstract

**Purpose:**

To investigate the correlation between postoperative feeding intolerance and defecation, with a view to carrying out prognostic assessment and timely intervention for the recovery of postoperative gastrointestinal function.

**Methods:**

The 114 neonates with congenital intestinal atresia who underwent primary anastomosis admitted to Shenzhen Children's Hospital from January 2014 to December 2022 were studied, and the patients' basic information, intraoperative conditions, postoperative feeding and defecation, and hospitalization time were retrospectively analyzed.

**Results:**

The risk factors for feeding intolerance after primary anastomosis for intestinal atresia are the gestational days, the time of the first postoperative defecations, the number of defecations on the previous day and the average number of defecations before feeding.

**Conclusion:**

The incidence of postoperative feeding intolerance is higher in preterm infants, and pediatricians can decide the timing of breastfeeding on the basis of the patients’ defecation. The focus on accurate defecation may be more meaningful in determining and predicting postoperative feeding intolerance in the infants.

## Introduction

With the advancement of medical technology and the emphasis on humanistic care, the concept of Enhanced Recovery After Surgery (ERAS) has been increasingly mentioned. A large number of evidence-based studies had pointed out that the incidence of postoperative problem and complications can be effectively reduced by regulating perioperative management measures, thereby alleviating the pain of children, promoting functional recovery, and reducing the hospitalization stays and costs [1, 2].

The congenital intestinal atresia presented as complete intestinal obstruction results in dilatation of the proximal bowel and disuse atrophy of the distal bowel [3]. Meanwhile, except for intestinal perforation, the uncontaminated abdominal environment permitted the choice of primary anastomosis in most cases of intestinal atresia [4]. Enteroanastomosis are well established even in general hospital, but the quick recovery of normal physiologic function has always been a difficult problem for pediatricians. And slow rehabilitation has also brought lasting mental pain and a huge economic burden to their families [5]. Feeding intolerance (FI) is a common complication of early enteral nutrition after gastrointestinal surgery, which refers to the patients presented with vomiting, stomach retention, bloating and other signs of intestinal obstruction in the process of the gradual increase of enteral feeding [6]. In this case, the pediatricians can only consider stopping the continuation of the increase in the amount of feeding or even temporarily suspend the feeding. There is no doubt that the occurrence of feeding intolerance can lead to the delay in reaching total enteral nutrition and prolonged hospital stays, and even cause malnutrition and growth restriction in newborns [7, 8].

In clinical practice, the postoperative defecation is an important reference for the pediatricians to decide to start feeding. At the same time, the record of defecation is more complete and objective than other physical signs. Therefore, this study retrospectively analyzed the clinical records of patients with congenital intestinal atresia who underwent primary anastomosis to investigate the correlation between postoperative feeding intolerance and defecation, with a view to carrying out prognostic assessment and timely intervention for the recovery of postoperative gastrointestinal function in children.

## Patients and methods

### Data collection

Approval was obtained from Ethics Committee of Shenzhen Children's Hospital (No. 202104202). We conducted a retrospective study of collected records from the neonates with congenital intestinal atresia who underwent primary anastomosis admitted to Shenzhen Children's Hospital between January 2014 and December 2022. Exclusion: unfaithful medical history, serious organic lesions other than digestive system, abdominal hemorrhage or trauma, hematological diseases such as leukemia, hemophilia, excessive resection of intestinal tubes resulting formation of short intestinal syndrome, anastomotic fistula or stenosis during the treatment period that seriously affected gastrointestinal function, and abandonment of the treatment because of various reasons. A total of 114 cases that finally met the inclusion and exclusion criteria were retrospectively collected based on the more credible parts of the medical records, including the patients' basic information, intraoperative situation, postoperative feeding and defecation, and hospital stays, etc.

### Postoperative feeding

Postoperative feeding after primary anastomosis follows the same feeding strategy in this single center institution. The pediatricians started enteral nutrition at the appropriate time based on the patients' abdominal symptoms, defecation, and abdominal X-ray, and gradually increased the same amount of feeds daily until complete elimination of intravenous nutrition was achieved. Feeding intolerance is defined as a delay or even temporary cessation of feeding due to intestinal obstruction such as vomiting, ventosity, bloody stools and so on during the process of increasing the feeding.

### Statistical analysis

Statistical analysis was performed with SPSS 26.0. Data conforming to normal distribution were expressed as mean (standard deviation), otherwise median (quartile), and categorical variables were calculated as frequency (percentage). Between-group comparisons between the two groups of feeding success and failure were performed by two independent-sample *t* test or chi-square tests, and meaningful risk factors were screened further for further multivariate logistic analysis.

## Results

All the differences between the two groups are shown in Table 1. As can be seen from the table, prognostic indicators such as postoperative complete enteral nutrition and hospital stays were significantly prolonged in the FI group compared to the feeding successful group. Meanwhile, the two groups had significant differences in gestational days, location of atresia, time of the first postoperative defecation, and the average times and amounts of defecation per day before starting feedings.

**Table 1:** The comparison between successful and unsuccessful feeding groups

Characteristics	Successful feedings	Feeding intolerance	Statistical value	*P* value
Female (female)	33 (38.4%)	14 (50.0%)	1.179	0.278
Age, days	1.43 ± 3.12	1.04 ± 1.75	0.636	0.526
Gestational days, days	262.22 ± 15.53	253.5 ± 14.87	2.607	0.010
Weight, g	2923.95 ± 558.08	2809.29 ± 574.38	0.938	0.350
Distance of intestinal atresia position from flexor ligament, cm	74.74 ± 43.01	52.19 ± 42.38	2.304	0.023
Length of resected bowel, cm	24.96 ± 12.9	23.12 ± 12.26	0.631	0.530
Time of first postoperative defecation, days	2.09 ± 1.06	3 ± 1.94	− 3.141	0.002
Time to start feeding, days	11.99 ± 6.4	13.5 ± 7.04	− 1.059	0.292
Average number of defecations per day before starting feeding	1.73 ± 0.98	1.17 ± 0.7	2.821	0.006
Average daily volume of defecation before starting feeding, g	6.76 ± 4.66	4.37 ± 2.99	2.539	0.013
Number of defecations on the previous day before feeding	2.02 ± 1.44	1.82 ± 1.7	0.616	0.539
Volume of defecation on the previous day before feeding, g	9.27 ± 7.87	7.79 ± 9.16	0.831	0.408
Hospital stay, days	28.94 ± 12.77	43.96 ± 15.84	− 5.087	< 0.001
Timing of full postoperative feedings, days	23.05 ± 10.46	39.93 ± 13.03	− 6.968	< 0.001

The relationship between all the influencing factors and feeding intolerance was analyzed by univariate logistic regression as shown in Table 2. Then the final independent risk factors were screened according to multivariate logistic regression: the time of the first postoperative defecation and the number of defecations on the previous day before feeding were risk factors for feeding intolerance, while the probability of feeding intolerance in patients with intestinal atresia may decrease with the increase in the gestational days and the average number of defecations per day before the start of feeding, as detailed in Table 3.

**Table 2:** Univariate logistic analysis of factors influencing postoperative feeding intolerance

Characteristics	*β*	Standard error	Wald	*P*	Exp(*B*) (95% Cl)
Gender	0.498	0.524	0.902	0.342	1.646 (0.589–4.600)
Age	− 0.061	0.118	0.265	0.607	0.941 (0.747–1.186)
Gestational days	− 0.044	0.025	3.182	0.074	0.957 (0.911–1.004)
Weight	0.001	0.001	3.116	0.078	1.001 (1.000–1.002)
Distance of intestinal atresia position from flexor ligament	− 0.002	0.009	0.049	0.825	0.998 (0.980–1.016)
Length of resected bowel	− 0.014	0.023	0.352	0.553	0.986 (0.943–1.032)
Time of first postoperative defecation	0.265	0.191	1.919	0.166	1.303 (0.896–1.895)
Time to start feeding	− 0.154	0.098	2.451	0.117	0.858 (0.708–1.039)
Total number of defecations before feeding	0.086	0.101	0.724	0.395	1.089 (0.894–1.327)
Total volume of defecations before feeding	0.008	0.022	0.132	0.716	1.008 (0.965–1.053)
Average number of defecations per day before starting feeding	− 1.965	1.299	2.287	0.13	0.140 (0.011–1.789)
Average daily volume of defecation before starting feeding	− 0.077	0.283	0.073	0.787	0.926 (0.532–1.613)
Number of defecations on the previous day before feeding	0.219	0.232	0.887	0.346	1.245 (0.789–1.962)
Volume of defecation on the previous day before feeding	− 0.03	0.047	0.404	0.525	0.970 (0.885–1.064)
Constant	9.605	5.491	3.06	0.080	–

**Table 3:** Multivariate logistic regression analysis of postoperative feeding intolerance

Characteristics	*β*	Standard error	Wald	*P*	Exp(*B*) (95%Cl)
Gestational days	− 0.032	0.019	2.664	0.103	0.969 (0.933–1.006)
Time of first postoperative defecation	0.385	0.211	3.337	0.068	1.469 (0.972–2.221)
Average number of defecations per day before starting feeding	− 1.127	0.510	4.873	0.027	0.324 (0.119–0.881)
Number of defecations on the previous day before feeding	0.470	0.235	4.006	0.045	1.599 (1.01–2.533)
Constant	6.837	4.947	1.91	0.167	−

## Discussion

With the aim of investigating the factors influencing postoperative feeding intolerance in children with intestinal atresia, we analyzed the data related to the admission and postoperative period of neonates who underwent primary anastomosis for congenital intestinal atresia, to provide more references to feeding decisions for pediatricians.

The time of the first flatus and defecation is an important indicator for assessing the recovery of gastrointestinal function of the patient after surgery [9–11]. In clinical practice, patients after gastrointestinal surgery usually need to resume anal flatus and defecation before eating. Van Bree S H et al. showed that the time to tolerance of solid food and having had defecation best reflects recovery of gastrointestinal function in the postoperative period [9]. In our study, there was a more significant correlation between the time of beginning postoperative feeding and the time of full feeding and the time of the first postoperative defecation, with the risk of feeding intolerance being greater for children whose first postoperative defecation occurred later. Furthermore, early feeding also promotes earlier recovery of gastrointestinal function, and faster attainment of full feeding means shorter time to hospital discharge [12–14]. The time to reach full feeding was 23.05 and 39.93 days for the successful and unsuccessful feeding group, respectively; this difference needs to be considered in the context of the effect of the time of the first defecation, the frequency and the amount of defecation on the feeding intolerance, and the discrepancy of 16 days between the two groups is more indicative of the effect of the feeding intolerance on the time to reach full feeding.

Compared to term infants, preterm infants undergoing neonatal surgery have the poorer prognosis and the higher mortality rate because of the immaturity of their organs and systems [15]. Functional peristalsis of the small intestine occurs in the fetus in the 30th week of gestation, and regular mobile compound motility occurs in the 33rd to 34th week of gestation, but gastrointestinal hormones do not reach normal levels until full term [16]. Therefore, gastrointestinal motility of preterm infants is significantly lower than that of full-term infants, which makes them more susceptible to intolerance symptoms such as vomiting and ventosity during the feeding [17]. In this study, gestational days was negatively correlated with the duration of feeding intolerance, which may be caused by a combination of insufficient gastrointestinal hormone secretion, underdeveloped swallowing, and immature intestinal motility in preterm infants.

The total times and the volume of defecations prior to initiation of feeding were also correlated with the time of initiation of feeding in the postoperative period, which is in line with the long-standing decision of clinicians to decide on the timing of breastfeeding initiation based on the patients’ defecation. The average number of defecations per day before starting feeding was negatively correlated with the feeding intolerance. Children who had more frequent defecations after the surgery had better peristalsis and patency, which reduced the likelihood of vomiting and bloating, and thus significantly reduced the odds of feeding intolerance. Interestingly, the opposite result was observed for the number of defecations on previous day of feeding. On the one hand, this result reflected the fact that pediatricians decide whether to start feeding based on the defecation of the children on the previous day in practice, and on the other hand, it may indicate that the frequency of defecation in children with feeding intolerance is overly concentrated in the period before feeding. In this way, paying attention to the defecation throughout the postoperative period may be more meaningful in determining and predicting the postoperative feeding.

In conclusion, feeding intolerance after primary anastomosis for neonatal intestinal atresia was associated with gestational days, time of first defecation, average number of defecations per day before starting feeding, and number of defecations on the previous day before feeding. Attention to more accurate information about defecation may be more valuable in determining and predicting postoperative feeding intolerance in infants.

## Data Availability

The data in this study is available for the public.
